# Lifetime of actin-dependent protein nanoclusters

**DOI:** 10.1016/j.bpj.2022.12.015

**Published:** 2022-12-14

**Authors:** Sumantra Sarkar, Debanjan Goswami

**Affiliations:** 1 The Center for Nonlinear Studies, Los Alamos National Laboratory, Los Alamos, New Mexico; 2 Theoretical Biophysics (T-6) Group, Los Alamos National Laboratory, Los Alamos, New Mexico; 3 Department of Physics, Indian Institute of Science, Bengaluru, Karnataka 560012, India; 4 NCI RAS Initiative, The Cancer Research Technology Program, Frederick National Laboratory, Frederick, Maryland

## Abstract

Protein nanoclusters (PNCs) are dynamic collections of a few proteins that spatially organize in nanometer-length clusters. PNCs are one of the principal forms of spatial organization of membrane proteins, and they have been shown or hypothesized to be important in various cellular processes, including cell signaling. PNCs show remarkable diversity in size, shape, and lifetime. In particular, the lifetime of PNCs can vary over a wide range of timescales. The diversity in size and shape can be explained by the interaction of the clustering proteins with the actin cytoskeleton or the lipid membrane, but very little is known about the processes that determine the lifetime of the nanoclusters. In this paper, using mathematical modeling of the cluster dynamics, we model the biophysical processes that determine the lifetime of actin-dependent PNCs. In particular, we investigated the role of actin aster fragmentation, which had been suggested to be a key determinant of the PNC lifetime, and we found that it is important only for a small class of PNCs. A simple extension of our model allowed us to investigate the kinetics of protein-ligand interaction near PNCs. We found an anomalous increase in the lifetime of ligands near PNCs, which agrees remarkably well with experimental data on RAS-RAF kinetics. In particular, analysis of the RAS-RAF data through our model provides falsifiable predictions and novel hypotheses that will not only shed light on the role of RAS-RAF kinetics in various cancers, but also will be useful in studying membrane protein clustering in general.

## Significance

Spatial organization of biomolecules shapes the behavior of a cell. It is particularly important during cell signaling, where transient, dynamic organization of the biomolecules helps cells process signals and respond to them. Nanoclusters, a specific form of dynamic organization of biomolecules, of peripheral membrane proteins, such as KRAS, play a critical part in the modulation of cell signals that control various cellular behaviors including cell growth, proliferation,and differentiation. Although we have made significant progress in understanding the structure, size, and origin of the nanoclusters, very little is known about the biophysical processes that control their lifetime. In this paper, we present a mathematical framework that provides quantitative insights into these processes and explains how oncogenic mutations in KRAS may lead to cancers.

## Introduction

Protein nanoclusters (PNCs) are dynamic collections of a small number of proteins that spatially organize in nanometer-length clusters (1,2,3,4,5,6,7,8,9,10). PNCs are one of the principal forms of spatial organization of membrane proteins, and they have been shown or hypothesized to be important in various cellular processes. In particular, it has been postulated that PNCs can digitize noisy analog signals that improve the signal/noise ratio of the signals received by a cell (11,12,13,14,15). Furthermore, it has been shown theoretically that PNCs can drastically improve the reaction rates of double-modification networks by allowing rapid multiple rebinding of an enzyme and its substrate (16). Importantly, the presence or absence of PNCs has measurable impact on the cell physiology and cellular behavior. For example, in mast cells, which control the response to allergic reactions, proliferation of the Fc ε R receptor clusters has been linked with the degranulation of the cells and strong allergic response (17). In another example, the formation of glycosylphosphatidylinositol-anchored protein clusters has been shown to be important in mechano-sensing and cell spreading (18). Therefore, understanding the dynamics of formation, growth, function, lifetime, and disintegration of PNCs is of paramount importance in our pursuit to understand and control cell signaling and the various diseases that it engenders.

Depending on the specific function and the cell type, the composition, size, shape, and the lifetime of the PNCs vary a lot. They can be homogeneous in composition, e.g., Kirsten rat sarcoma virus (KRAS) protein nanoclusters (3), or heterogeneous, such as in focal adhesion clusters (18,19). The shape can be isotropic, as of KRAS (4), or anisotropic, as of Harvey RAS PNCs (20). The number of proteins in a PNC (cluster size) also varies over quite a range. For example, KRAS nanoclusters typically contain 3–8 proteins (3,4), whereas Fc receptors can form clusters of 20–30 proteins (17). Similarly, the cluster radius can also vary from 20–200 nm (9). Finally, the lifetime and the stability of the clusters can also vary over a broad range: KRAS clusters, which are peripheral membrane protein clusters, are transient and survive for around 0.1–1 s (3,4), whereas Fc receptor clusters, which are integral membrane protein clusters, are stable and can survive the entire duration of the experiments (minutes) (17). Protein-protein interactions and protein-lipid interactions play a key role in determining the size, shape, composition, and stability of the PNCs specific to a biological process. Therefore, to understand the dynamics of PNCs in specific processes and cell types, several studies have investigated the underlying protein-protein and protein-lipid interactions (4,9). Despite the diversity of the PNCs and the underlying systems, these studies have shown that the formation of the PNCs can be categorized into actin-dependent and actin-independent groups (9), which provides a general framework for studying the dynamics of the PNCs. In this paper, we focus on the dynamics of actin-dependent peripheral membrane protein clusters.

**Figure 1:**
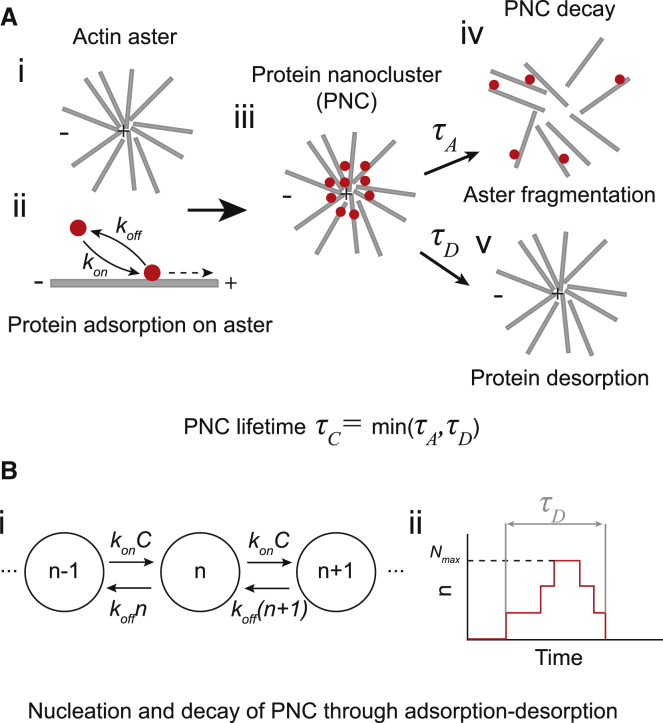
The aster-driven nucleation of protein nanoclusters. (*A*) (i) Dynamic cortical actin fibers form asters on the cell membrane. (ii) Peripheral proteins bind (adsorb) to the dynamic actin fiber with rate $k_{on}$ and are advected to the “+” end of the fiber. Once bound, it dissociates (desorbs) from the membrane with rate $k_{off}$. (iii) Proteins adsorbed on the membrane and advected to the plus end of the aster form a protein nanocluster. The protein nanocluster disintegrates either through (iv) the fragmentation of the aster with timescale $\tau_{A}$ or (v) through the desorption of all the proteins with timescale $\tau_{D}$. The lifetime of the cluster is the minimum of these two times. (*B*) The cluster formation model. (i) The cluster size n grows with rate $k_{on}C$ where *C* is the local concentration of actin in the aster, and decays with rate $k_{off}\times n$. (ii) The cluster grows from n=0 to a maximum size $n=N_{\max}$ and then decays back to n=0, after time $\tau_{D}$. We define $N_{\max}$ as the cluster size. To see this figure in color, go online.

The formation of the actin-dependent peripheral membrane protein clusters (PNCs henceforth) happens through a set of biomolecular processes that can be abstracted into a simple physical model, first developed to understand actin-dependent clustering of glycosylphosphatidylinositol-anchored protein (21). In this model, described in Fig. 1, the formation of actin asters aids the PNC formation. In particular, it is assumed that the protein adsorbs on the actin fiber with a rate $k_{on}$, advected to the aster center, and desorbs with a rate $k_{off}$, which leads to the formation of a dynamic PNC. Importantly, the protein absorption kinetics does not impact the actin self-organization dynamics in any way, but the actin asters do impact the PNC lifetime through the fragmentation of the asters. One corollary of these assumptions is that the aster lifetime solely determines the PNC lifetime. However, experimental observations do not corroborate this statement. In particular, recent experiments have revealed that asters survive for 10–500 s in in vivo conditions, and their typical fragmentation times are around 20 s (22,23). In contrast, many actin-dependent clusters, such as KRAS clusters, survive for only 0.1–1 s (4), which suggests that the lifetime of a PNC is determined by multiple physicochemical processes besides the aster fragmentation. Indeed, a prior work has suggested that stochastic protein absorption kinetics can be one such mechanism (24). However, it remains unclear whether the PNC lifetime is always determined by the stochastic growth kinetics or whether it is determined by both actin fragmentation and adsorption kinetics, depending on the specific situation. In this paper, we propose a model of PNC growth kinetics that, to the best of our knowledge, allows us to answer this question for the first time. Our model shows that although the formation of actin asters is necessary for the formation of actin-dependent clusters, under most biological conditions, they do not determine the lifetime of PNCs.

As an application of the general model presented in this paper, we also investigate the formation of heterogeneous protein clusters, such as the KRAS-RAF1 clusters, and their lifetime through a simple extension of this model. KRAS4B (RAS henceforth) is a peripheral membrane protein in human cells that interacts with the kinase RAF1 (RAF henceforth) to control cellular growth, differentiation, and proliferation (25,26,27). Mutated RAS and RAF is the underlying cause of 30% of all known cancers (28,29). In this paper, through the application of our general model, we have shown how oncogenic mutations in RAS lead to cancers. The insights gained from our quantitative predictions may be useful in developing therapeutics against these cancers.

## Materials and methods

### Experimental materials and methods

#### Cell culture, transfection and labeling of HaloTag-RAS/SNAP Tag-RAF

Mouse embryonic fibroblast (MEF) cells were genetically modified to express SNAP Tag fusion RAF1 and HaloTag fusion RAS protein (Fig. S1 *A* and *B*). Fusion constructs were incorporated in cells using viral delivery methods and selected with antibiotics for stable expression. MEF cells were plated and grown in six-well plates without antibiotics for 48 h before imaging. On the day of imaging, coverslips were washed with phosphate buffer saline, and cells were labeled with 100uM of fluorescent dye (SiR647), a cell permeable SNAP-tag ligand, which covalently binds to the SNAP Tag-RAF1 molecules. Cells were never fixed nor permeabilized for labeling. Fluorescently labeled SNAP-Cell 647-SiR (Cat #S9102S) ligands were obtained from New England Biolab. This fluorescence dye is highly photostable and resistant to photobleaching (Fig. S1 *C*) (30).

#### Single-molecule microscopy

Single-molecule imaging was carried out on the Nikon N- STORM microscope equipped with an APO ×100 TIRF objective of 1.49 NA (Nikon, Japan). A Tokai hit stage incubator (Tokai Hit, Japan) was used to provide 5% CO_2_ while maintaining the temperature at 37° for live cells. Labeled molecules (with SiR 647) associated with membrane were illuminated under TIRF mode. Then the JF646 dyes were excited with the 647-nm laser line, which is one of the four laser lines from the Agilent laser module of the Nikon N-STORM system (31)). The output laser beam was coupled into the Nikon TIRF box through a single mode fiber and focused into the back focal plane of the objective to form a parallel beam for wide-field operation. The TIRF illumination was achieved by changing the illumination angle through the Nikon TIRF box controlled by the Nikon software (NIS, Elements AR 4.4). Fluorescent signals from each molecule were recorded with a thermoelectric-cooled EM-CCD camera with 16-μm pixel size (iXon Ultra DU-897, Andor Technologies, USA) (Fig. S1 B). Single molecule tracking was implemented by time-lapse imaging of the molecules under continuous illumination at 10 ms exposure for a total of up to 2000 frames with zero delay time between frames. At this frame rate, membrane-bound molecules appear as transient, diffraction-limited fluorescence spots. An area of 16 × 16 μm^2^ of the plasma membrane in the cytoplasmic region of each cell was imaged (Fig. S1 *D*).

#### Single-molecule tracking data processing

The ImageJ-based single-molecule tracking plugin TrackMate (32) was used to create tracks from the time-lapse movies (Fig. S1 *D*). The single-molecule spot detection and tracking parameters were kept consistent across all experiments. These tracks were exported for residence time analysis using Matlab script (Mathwork, Natick, MA). Tracking data was obtained in multiple replicates for each and every condition (∼20,000 tracks and 20 cells). Residence time was calculated from each track using TrackArt (https://pubmed.ncbi.nlm.nih.gov/24885944/) software.

### Theoretical methods

#### Mathematical model of protein nanoclusters

The Langmuir kinetics (33) of protein adsorption-desorption can be summarized through the following reactions:

1. Adsorption of a protein and assimilation to the cluster of size n, denoted $P_{n}$ with rate $k_{on}$. We assume that the concentration of the unbound protein is much larger than the bound protein. Furthermore, we assume that the protein bound to actin does not affect its underlying dynamics (21). Hence, the propensity of adsorption is $k_{on}C$, where C is the *local* concentration of actin.

(1) $$P_{n}\overset{k_{on}}{\to }P_{n+1}$$

2. Desorption of a protein from a cluster of size n with rate $k_{off}$ and propensity $k_{off}n$.

(2) $$P_{n}\overset{k_{off}}{\to }P_{n-1}$$

#### Mathematical model of ligand-protein interaction

1. Adsorption of a ligand to a protein with rate $k_{1}$, which for a cluster of size n, happens with intensity $k_{1}n$. We again assume that the concentration of the unbound ligand is much higher than the bound ligand. The adsorption of a ligand changes the size of the ligand cluster of size m, $L_{m}$, by 1.

(3) $$L_{m}P_{n}\overset{k_{1}}{\to }L_{m+1}P_{n}$$

2. A ligand desorbs from a ligand cluster of size m with rate $k_{2}$ and propensity $k_{2}m$.

(4) $$L_{m}P_{n}\overset{k_{2}}{\to }L_{m-1}P_{n}$$

3. A ligand-protein complex desorbs with rate $k_{off}$ and propensity $k_{off}n$ to reduce both the ligand and the protein cluster size by 1.

(5) $$L_{m}P_{n}\overset{k_{off}}{\to }L_{m-1}P_{n-1}$$

#### Stochastic simulation of the mathematical models

**Figure 2:**
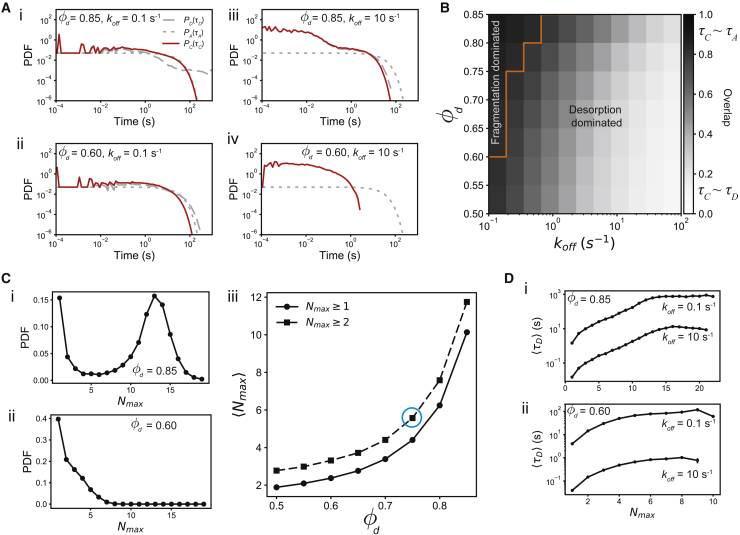
Protein nanocluster lifetime distribution. (*A*) Cluster lifetime (*solid red line*) and desorption time (*gray dashed line*) for different values of $\varphi_{d}$ and $k_{off}$. For all simulations, we assume that the aster lifetime is exponentially distributed with mean lifetime of 20 s (*gray dotted line*). The cluster lifetime is more similar to the aster lifetime when $\varphi_{d}$ is high and $k_{off}$ is low (i, ii), because for such parameters, the cluster is extremely stable and cluster decay happens because of aster fragmentation. Similarly, when $\varphi_{d}$ is low or $k_{off}$ is high (iii, iv), the cluster lifetime is more similar to the desorption timescale, because the clusters are much less stable. The similarity of $P\left(\tau_{C}\right)$ with $P\left(\tau_{A}\right)$ or $P\left(\tau_{D}\right)$ can be measured by finding the overlap between these distributions. For example, in (*B*) we show the overlap between $P\left(\tau_{C}\right)$ and $P\left(\tau_{A}\right)$, which consolidates our observation that cluster lifetime is similar to aster lifetime when $\varphi_{d}$ is high and $k_{off}$ is low. The orange line marks the boundary where the overlap of $P\left(\tau_{C}\right)$ with $P\left(\tau_{A}\right)$ is higher than that with $P\left(\tau_{D}\right)$. (*C*) The distribution of cluster size, $N_{\max}$, depends on $\varphi_{d}$, but not on $k_{off}$ (not shown). The distribution for (i) $\varphi_{d}=0.85$ and (ii) $\varphi_{d}=0.60$ shows that as $K_{d}$ increases, the distribution develops a peak at $N_{\max}>1$, which leads to the nonlinear increase of the average $\left\langle N_{\max}\right\rangle$ with $\varphi_{d}$. (*D*) This change in the cluster size distribution drastically impacts the average desorption time $\left\langle \tau_{D}\right\rangle$. When (i) $\varphi_{d}=0.6$, the average lifetime grows beyond the average aster lifetime, $\left\langle \tau_{A}\right\rangle \text{,}$ only when $k_{off}$ is small, but (ii) for $\varphi_{d}=0.85$, the average lifetime grows rapidly beyond $\left\langle \tau_{A}\right\rangle$. These two results reaffirm the location of the boundary in (*B*). The blue circle in (*C*) (iii) indicates the value of $\varphi_{d}$ that is most similar to experimentally observed RAS cluster size distribution. $10^{4}$ independent realizations of the model were used for these results. To see this figure in color, go online.

**Figure 3:**
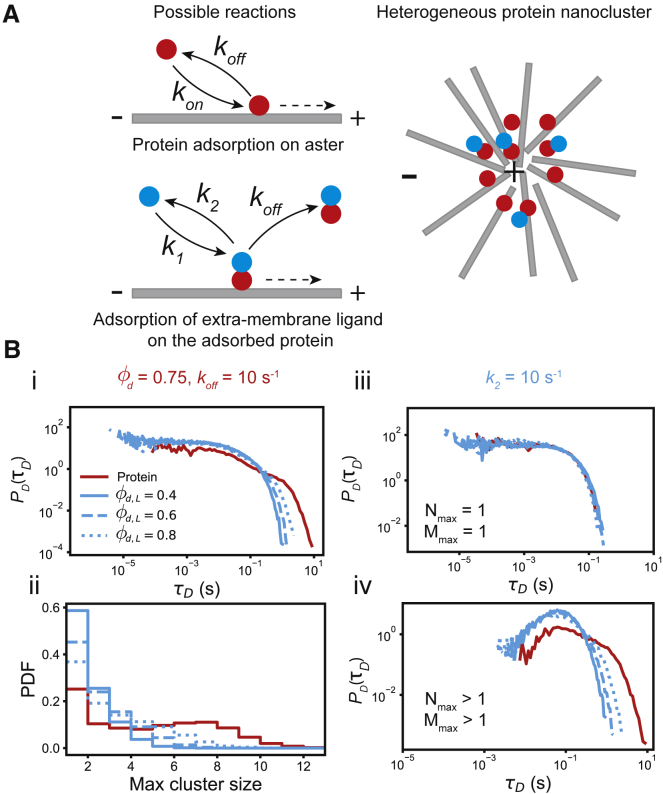
Ligand-protein interaction on protein nanocluster. (*A*) The model from Fig. 1*A* is amended to include interaction of the clustered protein target (*red*) with an extra-membrane ligand (*blue*). The ligand binds to the target with rate $k_{1}$ and dissociates with a rate $k_{2}$. Therefore, in the presence of n targets, the number of the ligand, m, grows with propensity $k_{1}n$ and decays with propensity $k_{2}m$. In addition, the ligand-target complex also dissociates with propensity $k_{off}m$. Collectively, these reactions lead to the formation of a heterogeneous cluster, with sizes $N_{\max}$ for the protein target (*red*) and $M_{\max}$ for the ligand (*blue*). (*B*) Because $\varphi_{d}=0.75$ and $k_{off}=10\;s^{-1}$ fall in the desorption dominated region in Fig. 2*B*, the cluster lifetime is almost entirely determined by the desorption timescale $\tau_{D}$. In (i) we have plotted $P\left(\tau_{D}\right)$ for both the target and the ligand, and in (ii) we have plotted the cluster size distribution for various $\varphi_{d,L}$ values (legend). As evident from (ii), the clusters with size 1 contribute heavily to $P\left(\tau_{D}\right)$. To isolate the effect of cluster size, in (iii) we plot $P\left(\tau_{D}\right)$ for $N_{\max}=M_{\max}=1$, which decays exponentially with rate $k_{off}$, whereas in (iv), $P\left(\tau_{D}\right)$ for $N_{\max},M_{\max}>1$ have nonmonotonic distributions. $10^{4}$ independent realizations of the model were used for these results. To see this figure in color, go online.

The number of proteins in a cluster is very small (usually <30), so the kinetics becomes nondeterministic due to intrinsic noise originating from small copy number of the proteins. As a result, we cannot use ordinary differential equation-based chemical kinetic models and have to investigate these models using chemical master equations. The chemical master equations for the reactions described here were simulated using the Gillespie algorithm (34). To generate the distributions in Figs. 2 and 3, for each set of parameters, $10^{4}$ independent time series were generated by simulating the model using the Gillespie SSA. Each of these time series were run for a maximum of 1000 s or until the proteins completely desorbed from cluster, such that the cluster size became 0, whichever was shorter.

#### Distribution of $\tau_{C}$

The cluster lifetime $\tau_{C}$ is given by the minimum of the aster fragmentation time $\tau_{A}$ and the protein cluster desorption time $\tau_{D}$. That is $\tau_{C}=\min\left(\tau_{A},\tau_{D}\right)$. We assume that the probability density distributions $P_{A}\left(\tau_{A}\right)$ and $P_{D}\left(\tau_{D}\right)$ are nonidentical, but independent of each other because the aster fragmentation and the protein desorption are independent processes for the clustering proteins that we consider here. They will not be independent if the clustering of the proteins, e.g., Myosin or Arp2/3 complex, directly influences the formation of the asters.

To find the distribution of $\tau_{C}$, $P_{C}\left(\tau_{C}\right)$, we use the cumulative distribution function trick. To perform this trick, observe the following:

Obs 1: $\tau_{C}>\tau$ if and only if $\tau_{D}>\tau$ and $\tau_{A}>\tau$.

Therefore,

(6) $$P_{c}\left(\tau_{C}>\tau \right)=P\left(\tau_{D}>\tau \;and\;\tau_{A}>\tau \right)$$

Using the independence of the probability distributions of $\tau_{A}$ and $\tau_{D}$, and using the fact that the cumulative distribution function $F_{c}\left(\tau \right)=1-P_{C}\left(\tau_{C}>\tau \right)$, we can rewrite the above expression as follows:

(7) $$F_{c}\left(\tau \right)={1-P}_{D}\left(\tau_{D}>\tau \right)P_{A}\left(\tau_{A}>\tau \right)=1-\left[1-F_{D}\left(\tau \right)\right]\left[1-F_{A}\left(\tau \right)\right]$$

The distribution $P_{c}\left(\tau \right)=\frac{\partial F_{c}\left(\tau \right)}{\partial \tau }$. Therefore, the distribution is given by

(8) $$P_{c}\left(\tau \right)=S_{A}\left(\tau \right)P_{D}\left(\tau \right)+S_{D}\left(\tau \right)P_{A}\left(\tau \right)\text{,}$$

where $S_{X}\left(\tau \right)=1-F_{X}\left(\tau \right)$ is the survival function.

#### Measurement of overlap

We measured the overlap between two probability distributions using the Bhattacharya coefficient (BC) (35). The Bhattacharya distance $D_{B}$ (defined below) and the Kullback-Leibler divergence ($D_{KL}$) (36), which is usually used to measure the distance between two probability distributions, give quantitatively similar results. The advantage of the Bhattacharya distance is that it is symmetrical for both distributions. For two continuous probability density functions P and Q, these measures are defined as follows:

(9) $$BC\left(P,Q\right)=\int_{-\infty }^{\infty }\sqrt{P\left(x\right)Q\left(x\right)}dx$$

(10) $$D_{B}=-\ln\;BC\left(P,Q\right)$$

(11) $$D_{KL}\left(P\|Q\right)=\int_{-\infty }^{\infty }P\left(x\right)\log\left(\frac{P\left(x\right)}{Q\left(x\right)}\right)dx$$

#### Best fit distribution

**Figure 4:**
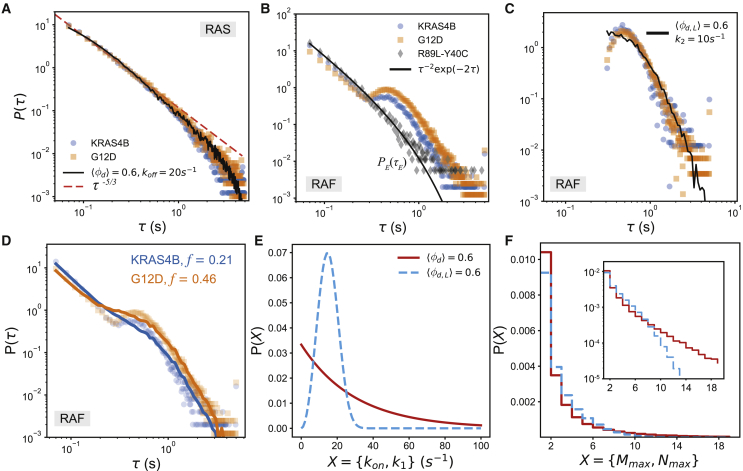
RAS-RAF interaction. (*A*) RAS residence time: experimentally observed residence time distributions of wild-type KRAS4B (*blue circles*) and KRAS4B with G12D mutation (*orange square*). The black line is the model prediction. The dashed red line shows the power law predicted by disordered systems theory (supporting material). (*B*) RAF residence time on the membrane in the presence of different RAS mutations (same color scheme as in *A*). Both distributions show power law decay at short times (τ<0.25s) and nonmonotonic decay at longer times. The power law is also observed in the presence of R89L mutation in RAS and Y40C mutation in RAF (*black diamonds*). These two mutations remove any interaction between RAS and RAF. Therefore, this observation suggests that the power law for short times arises due to RAF-membrane interactions, and the nonmonotonic decay at longer times arises due to RAS-RAF interactions. (*C*) Because these two processes are independent, we subtract the R89L-Y40C curve from the other two to get the RAF residence time distribution arising purely due to RAS-RAF interactions (*blue and orange markers*). We find good match with our theoretical predictions of $P\left(\tau_{C}\right)$ (*black curve*) when two conditions are met: 1) $k_{on}$ (*red* in *E*) and $k_{1}$ (*sky blue* in E) are randomly distributed, and 2) we consider the distribution of residence time only when $M_{\max}\geq 8$. (*D*) Adding our model’s prediction with the power law from membrane-RAF interaction reproduces (*solid lines*) experimental observations (*markers*) to an excellent degree. (*E*) The distribution of random $k_{on}$ and $k_{1}$ values used in the model with $\left\langle \varphi_{d}\right\rangle =0.6$, $\left\langle \varphi_{d,L}\right\rangle =0.6$, $k_{off}=20{\;s}^{-1}$, and $k_{2}=10\;s^{-1}$. $k_{on}$ is exponentially distributed, and $k_{1}$ is distributed as a Weibull distribution with shape parameter 3. The scale of both distributions is determined from the mean, $\left\langle \;k_{on\;\left(1\right)}\right\rangle$, which is a function of $\left\langle \varphi_{d\;\left(d,L\right)}\right\rangle$ and $k_{off\;\left(2\right)}$. (*F*) The cluster size distribution of RAS (*red*) and RAF (*sky blue*) predicted from our model. Inset: the same distribution in semilog scale. $10^{5}$ independent realizations of the model were used to obtain each distribution. To see this figure in color, go online.

To find the best fit distributions shown in Fig. 4 *C* and *D*, we minimized the distance between the experimentally observed distribution and the theoretical distribution obtained from the weighted sum of $P_{E}\left(\tau_{E}\right)$ and $P_{C}\left(\tau_{C}\right)$:

(12) $$P_{T}\left(\tau \right)=fP_{C}\left(\tau \right)+\left(1-f\right)P_{E}\left(\tau \right)$$

The distances were measured using the Bhattacharya distance $D_{B}$ and the KL divergence $D_{KL}$, both of which predicted identical values of $M_{th}$ and f.

## Results

### Determinants of protein nanocluster lifetime

To understand the processes that determine PNC lifetime, we used a simplified version of the model proposed by Gowrishankar et al. (21) In particular, we assume that the aster formation happens at a timescale much faster than the PNC lifetime, such that the aster has already reached its steady-state structure when the first protein adsorbs on the aster. This assumption decouples the transient PNC kinetics from the transient aster dynamics, and we can incorporate the contribution of the aster on the PNC kinetics simply through the local actin concentration C. Under this assumption, the PNC lifetime is determined by two processes: 1) the stochastic growth and decay of the PNC through protein adsorption and desorption, which gives a desorption time $\tau_{D}$, and 2) aster fragmentation with mean fragmentation time $\tau_{A}$ (Fig 1 *A*). The cluster lifetime $\tau_{C}$ is given by the shorter of these two times. That is,

(13) $$\tau_{C}=\min\left(\tau_{D},\tau_{A}\right)$$

To better understand the origin of $\tau_{D}$, consider the model shown in Fig 1 *B* (i). The proteins adsorb onto the aster at a propensity $k_{on}C$, which increases the size of the cluster by one protein. The size of the cluster decreases through the desorption process, which, for a cluster of size n, happens at a propensity $k_{off}n$. We also assume that all asters are identical, such that they have identical actin concentration C, which allows us to absorb C in $k_{on}$, so the propensity of growth is $k_{on}$. We relax this assumption later to include variable C also. Due to the small number of proteins, the growth and decay of the cluster is a stochastic process in which the size of the cluster grows from n=0 to a maximum size $N_{\max}$ and then ultimately decays back to n=0. We define the total time between these two n=0 states as $\tau_{D}\text{,}$ and the cluster size is given by $N_{\max}$ (Fig 1 *B* (ii)).

To understand the relative contribution of the stochastic growth and the aster fragmentation on the lifetime of a PNC, we measured the distribution of $\tau_{D}$, $P_{D}\left(\tau_{D}\right)\text{,}$ by varying $k_{off}$ and the duty ratio $\varphi_{d}=\frac{k_{on}}{k_{on}+k_{off}}$, which measures the fraction of time a single protein remains bound to the actin; for constant $k_{off}$, varying $\varphi_{d}$ is equivalent to varying $k_{on}$. Although we could have varied $k_{on}$ instead of $\varphi_{d}$, we find that the latter is a more natural candidate to describe binding kinetics. Indeed, $\varphi_{d}$ is exactly the probability of binding in the limit of high protein concentration (see supporting material). Following experimental observations (22,23,37), we assumed that the aster fragmentation time, $\tau_{A}$, is exponentially distributed with $\left\langle \tau_{A}\right\rangle =20\;s$. Given $P_{D}\left(\tau_{D}\right)$ and $P_{A}\left(\tau_{A}\right)$, the cluster lifetime distribution is given by the following:

(14) $$P\left(\tau_{C}\right)=S_{A}\left(\tau_{c}\right)P_{D}\left(\tau_{C}\right)+S_{D}\left(\tau_{C}\right)P_{A}\left(\tau_{C}\right)\text{,}$$

where $S_{A}$ and $S_{D}$ are the survival probability functions of $P_{A}$ and $P_{D}$ (see supporting material). This expression simply states that the cluster lifetime $\tau_{C}$ is determined by $P_{D}$, if the aster survives until time $\tau_{C}$, or else it is determined by $P_{A}$. In Fig. 2 *A*, we show some example distributions. When both $k_{off}$ is small and $\varphi_{d}$ (equivalently $k_{on}$) is large, a protein adsorbs at a rate much higher than it desorbs and once adsorbed takes a long time to desorb. We find that, in these situations, the cluster lifetime is determined by the aster fragmentation time (Figs. 2 *A* (i), (iii), and S2). In contrast, for all other situations, $P\left(\tau_{C}\right)$ is determined by $P\left(\tau_{D}\right)$ (Fig. 2 *A* (ii), (iv)). We can make this observation more quantitative by measuring the overlap between $P\left(\tau_{C}\right)$ and $P_{D}\left(\tau_{C}\right)$ or $P_{A}\left(\tau_{C}\right)$. In Fig. 2 *B*, we show the overlap between $P\left(\tau_{C}\right)$ and $P_{A}\left(\tau_{C}\right)$, which shows that protein desorption determines the cluster lifetime for a large set of parameters. Only when the adsorption-desorption process is extremely slow and the protein strongly adsorbs to the actin fiber, then the lifetime is determined by the aster fragmentation time. This observation shows that although actin aster is necessary for the formation of actin-dependent PNCs, in cellular conditions, its fragmentation may rarely determine the PNC lifetime. A simple testable prediction from this model is that, as the aster fragmentation time shortens, e.g., through the application of Latrunculin (2), there will be a sharp transition in the cluster lifetime distribution.

### Dependence of desorption time on the cluster size

The cluster size, i.e., $N_{\max}$, does not depend on the desorption rate $k_{off}$ and depends only on the duty ratio $\varphi_{d}$. As $\varphi_{d}$ increases, the cluster size distribution transitions from a unimodal distribution to a bimodal distribution, implying that larger clusters become more prevalent at higher $\varphi_{d}$ values. Indeed, the mean size of the clusters increases nonlinearly with $\varphi_{d}$, being close to 1 for $\varphi_{d}\approx 0.5$. The average desorption time, $\left\langle \tau_{D}\right\rangle$, has a nontrivial dependence on the cluster size and $k_{off}$. In particular, $\left\langle \tau_{D}\right\rangle$ versus $N_{\max}$ shows three different regimes. When $N_{\max}$ is small, $\left\langle \tau_{D}\right\rangle$ increases subexponentially with $N_{\max}$, followed by exponential increase, and saturation to a maximum value that depends on $\varphi_{d}$ and $k_{off}$. For example, for $\varphi_{d}=0.6$, $\left\langle \tau_{D}\right\rangle$ saturates to a value that is much smaller than the simulation ceiling (1,000 s), whereas for $\varphi_{d}=0.85$ and $k_{off}=0.1{\;s}^{-1}$, the saturation happens at the simulation ceiling, implying that the $\left\langle \tau_{D}\right\rangle$ values are much longer than the simulation ceiling. This observation also clarifies why we observe fragmentation-dominated cluster lifetime only when $k_{off}$ is small and/or $\varphi_{d}$ is large. As our results show, only in this limit are the cluster sizes large enough so $\tau_{D}$ is much longer than $\tau_{A}$.

### Ligand-protein interaction on protein nanoclusters

One recurrent feature of cell signaling systems is that cytosolic or extracellular ligands are recruited to a membrane PNC in response to a signal (38). For example, the effector protein RAF is recruited to clustered RAS, which starts a MAPK signaling pathway for cell growth and proliferation (25,26). Therefore, it is important to understand how the clustering of the membrane proteins influences and modulates the ligand dynamics. Investigation of this problem is particularly exciting when the membrane protein forms desorption-dominated PNCs, because in such a situation the ligands form a transient cluster whose growth kinetics are intimately coupled to the growth kinetics of the underlying PNC. For fragmentation dominated PNCs, because of their long lifetime, the ligand growth kinetics are very similar to the PNC kinetics. Because of this reason, in this paper, we are only going to focus on the ligand-protein interaction on desorption-dominated PNCs.

To model the ligand-protein interaction, we extend our model of PNC formation by including an additional molecular species (blue particles in Fig 3 *A*) that interacts only with the proteins (red particles in Fig 3 *A*) in the PNC. This new species, which is the ligand, adsorbs to a protein with a rate $k_{1}$ and desorbs with rate $k_{2}$, which together determine the ligand duty ratio $\varphi_{d,L}=\frac{k_{1}}{k_{1}+k_{2}}$. The ligand can also unbind from the membrane when the protein it is bound to desorbs from the actin aster. We have assumed that binding of the ligand to the protein does not change the protein’s desorption rate, so that the ligand-protein complex desorbs with rate $k_{off}$. Essentially, this model adds another layer of 1:1 Langmuir adsorption kinetics (33) on top of the protein-actin kinetics to understand the ligand-protein interactions on PNCs. Therefore, similar to the protein, the ligand can also form a cluster by adsorbing to the protein, which grows from m=0 to $m=M_{\max}$ at a propensity $k_{1}n$, where n is the instantaneous number of proteins in the PNC. The ligand cluster decays at a propensity $k_{2}m$ and eventually returns to the m=0 state, which again determines the desorption time of the ligand cluster. The ligand cluster lifetime is determined by three timescales: its own desorption time, the desorption time of the PNC, and the aster fragmentation time. The aster fragmentation time is unimportant for the desorption dominated PNCs, which allows us to infer the ligand lifetime solely from the ligand cluster and the PNC cluster desorption times.

The ligand-protein interaction varies widely depending on $\varphi_{d},k_{off},\varphi_{d,L}$, and $k_{2}$, some of which we have shown in Fig. S3. In the rest of the paper, we apply our general framework on the specific case of RAS-RAF interaction, which is a well-known model system. In particular, from biochemical measurements of the interaction between the Ras binding domain of RAF and RAS, it has been shown that RAF dissociates from RAS approximately at a rate of $k_{2}∼10\;s^{-1}$ (39). We also know that the average KRAS cluster contains about 6–8 proteins (3,4), which implies that in our model, $\varphi_{d}\approx 0.75$ (Fig. 2 *C* (iii)). Finally, we also know that typical KRAS clusters survive for 0.1–1 s (4), which we get when $k_{off}\;∼\;10\;s^{-1\;}$ (Fig. 2 *D*). Therefore, $\varphi_{d,L}$ is the only free parameter in our model. In Fig. 2 *B*, cases with different $\varphi_{d,L}$ are shown. Because binding of the ligand does not change the protein kinetics, changing $\varphi_{d,L}$ does not change the lifetime of PNC. In contrast, increasing $\varphi_{d,L}$ at constant $k_{2}$ increases the propensity of ligand binding, which increases the lifetime of the ligand cluster. These observations are reflected in $P\left(\tau_{D}\right)$: although the distribution remains unchanged for PNCs, for the ligands, the tails become broader with increasing $\varphi_{d,L}\text{.}$ Due to the same reason, the size distribution of protein cluster remains unaffected, but the ligand cluster becomes larger as $\varphi_{d,L}$ increases. Therefore, purportedly, the change in the ligand cluster lifetime distribution happens due to the increase in the number of ligand clusters with size greater than 1. Indeed, resolving the lifetime distribution by the size of the ligand ($M_{\max}$) and the protein clusters ($N_{\max}$) shows that the lifetime distribution of clusters of only one protein ($N_{\max}=1$) or ligand ($M_{\max}=1$) depends only on the desorption rates and remains unaffected by the variation of $\varphi_{d,L}$. In contrast, the lifetime of ligand clusters with $M_{\max}>1$, changes with $\varphi_{d,L}$. Therefore, the formation of ligand clusters on PNCs provides a mechanism to control the residence time of extra-membrane ligands.

### RAS residence time on the membrane

Due to their roles in various cancers, understanding RAS-RAF interaction has been subject of extensive investigations, where it has been reported that RAS forms actin-dependent PNCs on the inner leaflet of the plasma membrane only when it is in the GTP-bound active form (2,4,40). Also, it is well-known that RAF binds principally to GTP-bound RAS (27). Hence, it is likely that RAS-RAF interaction in human cells is mediated by RAS nanoclusters. Therefore, RAS-RAF interaction provides an excellent experimental platform for understanding the kinetics of ligand-cluster interaction on real PNCs.

To do so, we measured the residence times of RAS and RAF on MEF cell membranes using TIRF microscopy and single-particle tracking (Fig. S1). To understand the effect of interaction between RAS and RAF on the residence time of RAF on the membrane, we used wild-type (wt) and mutated variants of RAS and RAF. The residence time of RAS decays as a power law with an exponential tail (Fig. 4 A). This distribution is qualitatively similar to the distribution predicted by our model (Figs. 2 *A* and 3 *B*), except that our model predicts an exponential decay of the residence times, whereas we get a power law tail here. This quantitative difference can be explained by noting a key difference between our model and the cellular systems. In the model, we assumed that the actin concentrations are identical in all asters, which does not apply to a cellular system, where the cortical actin concentration can vary substantially over space and time (37).

A simple way to incorporate the variation of actin concentration in our model is to assume that $k_{on}$ varies randomly from aster to aster due to spatial variation in C, but it remains constant for an aster. An argument from the physics of disordered systems proposes that if a timescale τ is determined by some underlying variable E, then the distribution of the timescale is determined by the distribution of the disorder (41). In particular, if $\tau =e^{E/E_{1}}$ and $P\left(E\right)=\frac{1}{E_{0}}e^{-E/E_{0}}$, then $P\left(\tau \right)\propto \tau^{-1-\frac{E_{1}}{E_{0}}}$. Indeed, we find that $\left\langle \tau_{C}\right\rangle$ varies exponentially with $k_{on}$. Also, experimental observations suggest that aster size (hence, actin concentration C) also has exponential tails (Fig. S4). Hence, following the physical argument, we can immediately see that $P\left(\tau_{C}\right)$ for RAS should have power law tails. In particular, we get an excellent fit to the experimental distribution when $k_{on}$ varies exponentially (Fig. 4 *E*) with $\left\langle k_{on}\right\rangle$, the only parameter of the distribution, determined by $k_{off}=20{\;s}^{-1}$ and $\left\langle \varphi_{d}\right\rangle =0.6$ (Fig 4 *A* and S5). Interestingly, the obtained value of $k_{off}$ is remarkably close to the observed GTP hydrolysis rate of RAS GTPase activating proteins (42).

### RAF residence time on the membrane

The residence time of RAF on the membrane also follows a nonexponential distribution (Fig. 4 *B*–*D*), which suggests that the residence time of RAF is “not” determined by uncorrelated collisions with the membrane, and the interactions of RAF with RAS and the membrane are its important determinants. Indeed, upon closer inspection of the residence time distribution for wt.RAS and RAF (Fig. 4 *B*) we found that the residence time, τ, has two unique regimes: for τ<0.25s, the distribution decays as a power law, and above this timescale, it decays nonmonotonically (has a peak) with a different power law tail. To understand the origin of these two regimes, we repeated the experiments on RAS with R89L and RAF with Y40C mutations. The mutations eliminate any interaction between RAS and RAF. For this system, the nonmonotonic part disappeared from the residence time distribution, and only the power law decay remained (Fig. 4 *B*), which implies that the nonmonotonic part originates from the RAS-RAF interactions, whereas the initial power law decay originates from the RAF-membrane interaction. This is further evinced by the residence time distribution of RAF in the presence of RAS with G12D mutations, which increases the fraction of GTP-bound RAS. In this system, the peak of the nonmonotonic part becomes more pronounced (Fig. 4 *B*).

The above observations show that the initial power law and the nonmonotonic decay at later times originate from two independent processes. Hence, we can isolate the contribution of RAS-RAF interaction on the RAF residence time by subtracting the power law obtained from R89L-Y40C system from the other two residence time distributions. Doing so produces the distributions shown in Fig. 4 *C*. The distributions are identical to each other within experimental variations and have power law tails that decay as $\tau^{-3.5}$. Remarkably, the shape of the distributions is qualitatively similar to the distribution of ligand cluster desorption times (Fig 3 *B*). Hence, it is possible that the nonmonotonic distribution arises due to the formation of RAF clusters on the RAS clusters. However, it is also possible that the nonmonotonicity arises due to the multiple rebinding of a single RAF on the cluster, which increases its lifetime and leads to a nonexponential and nonmonotonic distribution. However, our computational results showed that although multiple rebinding of a single ligand increased the residence time, it did not make the residence time distribution nonmonotonic (Fig. S6), which ruled out multiple rebinding as a possible origin of the observed nonmonotonic distribution. Importantly, this result established that ligand clustering is the underlying mechanism of the nonmonotonic decay of residence times. More important, the identity of the distributions implies that the binding affinities of RAF ($\varphi_{d,L}$) are identical for both wt and mutant RAS.

Similar to the RAS residence time distribution, the power law decay in RAF residence time distribution can be explained by the spatial variation of $k_{1}$ from one PNC to another because of the differences in local lipid environments or variation in effective reaction rates due to competition from other interaction partners of RAS. We incorporate the spatial variation by assuming that (besides $k_{on}$) $k_{1}$ is also randomly distributed and it follows a Weibull distribution with shape parameter 3 (Fig. 4 *E*). Unlike the RAS distribution, there is no observational evidence for this distribution, but there is a physical argument, which we discuss in the supporting material. Remarkably, we find that introduction of the random $k_{on}$ and $k_{1}$ suffices to reproduce the power law tail. In fact, we can quantitatively reproduce the distribution when we consider the lifetime of ligand clusters with size greater than a cutoff $M_{th}=8$, i.e., $M_{\max}\geq M_{th}=8$ and $\langle \varphi_{d,L}\rangle =0.6$ (Figs. 4 *C*, *D*, and S7), the latter of which uniquely determines the scale of the Weibull distribution from which $k_{1}$ is sampled. This result confirms that the nonmonotonicity arises solely because of the formation of RAF clusters on RAS clusters. Indeed, the cluster size distribution predicted by our model (Fig. 4 *F*) is consistent with prior experimental (4,20) and computational observations (20,43,44,45).

### Impact of the G12D mutation of RAS

Next, using our model, we investigate the origin of the difference between the residence time distributions in the presence of wt and G12D RAS. We ask, why do we see an increase in the nonmonotonic part of the distribution in the presence of G12D, even though the RAS-RAF binding affinity remains unchanged? In our model, in the absence of any changes in the binding affinity, the nonmonotonic part can become more prominent if and only if the clustered fraction of RAF increases by binding to RAS PNCs. To test this hypothesis, we added the experimentally obtained power law $P_{E}\left(\tau_{E}\right)$ (Fig 4 *B*) to the cluster time distribution $P_{C}\left(\tau_{C}\right)$ from our model and generated a combined distribution by taking a weighted mean of the two distributions. The resultant distribution is

(15) $$P_{T}\left(\tau \right)=\left(1-f\right)P_{E}\left(\tau \right)+fP_{c}\left(\tau \right)\text{,}$$

where f is the fraction of RAF that binds to RAS PNCs. We found a best fit distribution for both cases by varying the threshold $M_{th}$ (Fig. S7) and f. Remarkably, we found that both best fit distributions had identical $M_{th}=8$, but the f values were different by a factor of two (Fig 4 *D*). This result implies that, in the timescales probed by our experiments (∼5 s), the G12D mutation does not change the binding affinity between RAS and RAF, but it increases the number of RAS clusters. This result is consistent with the experimental observation that only RAS.GTP forms clusters (4). Indeed, as noted earlier, the best fit $k_{off}$ value is remarkably close to the GTP hydrolysis rate of GTPase activating proteins, which implies that RAS.GTPs are the drivers of the clustering. Because the G12D mutation effectively increases the number of RAS.GTP on the membrane, it is likely that we will observe more RAS clusters in the presence of this mutation.

## Discussion

In this paper, we have presented a simple mathematical model to understand the lifetime of actin-dependent peripheral membrane PNCs and protein-ligand interactions on the PNCs. Our results show that in many biologically relevant cases, the lifetime of PNCs is determined solely by the adsorption-desorption kinetics of the proteins on actin asters and not by the fragmentation of the aster. Our model shows that many PNCs arise from subcritical nucleation (46) of peripheral membrane proteins that survive for a short time before disintegrating. Under special circumstances, the nucleus becomes large enough to be stable and survives for a long duration. Only in these cases, the fragmentation of the aster determines the lifetime of the PNC. Therefore, from a physical standpoint, the dynamics of PNCs is better understood by studying the dynamics of subcritical nuclei, as we have done in this paper.

To understand the effect of protein clustering on the ligand-protein interactions, we studied some ideal cases using our model, which showed that the clustering of the proteins on the membrane enhances the residence time of the ligands on the membrane (Fig. 3 *B* (iii), (iv)). After incorporating the effect of a spatially heterogeneous membrane in our model, we compared our results with experimental measurements of RAF residence times in the presence and absence of its interaction with the PNC forming RAS protein. We found remarkable agreement between our predictions and the experimental observations, which consolidated the results obtained from our model. Importantly, investigation of this system using our model allows us to contribute to an ongoing debate on the role of RAS G12D mutation in the proliferation of cancer cells. Our model suggests that the G12D mutation does not change the binding affinity between RAS and RAF. Instead, it increases the propensity of RAS and RAF cluster formation, which increases the residence time of RAF on the membrane and enhances the activity of the MAPK pathway involved in proliferation. An independent experiment on HeLa cells shows very similar results (not shown), which gives further credence to our proposition.

Although our model showed remarkable agreement between the experimental observations, there are several drawbacks that need to be addressed to develop a better model of the PNCs. We assumed well-mixed mass action kinetics, which is likely to be invalid in most biological contexts (47). The well-mixed kinetics also overestimate the frequency of events happening at short times, because of which our model disagrees with the experimental observation at short times (Fig 4 *B*–*D*). Also, in modeling the protein-ligand interactions, we did not explicitly model the multiple rebinding of a ligand on a PNC and assumed that it will be captured by some effective $k_{on}$ or $k_{1}$ values. To our satisfaction, recalculation of the results by adding a well-mixed model of multiple rebinding does not change our results qualitatively (not shown). Furthermore, the lipid environment of proteins plays an important part in determining the stability of the PNCs (4,9,43,48,49), which we do not consider here explicitly, but model as a source of spatial heterogeneity in the rates. In the future, we will develop models to incorporate these features. Despite these limitations, our current model provides deep insights into the working of the PNC formation and lifetime that will be useful in our understanding of protein nanoclusters and protein-protein interactions during cell signaling.

In conclusion, in this paper, we present a general, yet simple, framework to study protein nanocluster dynamics in spatially heterogeneous cell membranes. As demonstrated here, through this framework, we can not only study general questions regarding the growth and stability of protein nanoclusters, but also apply them to study specific protein-protein interaction systems. The framework presented is not specific to the RAS-RAF system and can be used to model other protein-protein interactions just by varying the parameters. Furthermore, with little modification, our framework can be used to understand drug-protein interactions, which may be useful in rapid design of novel drugs. We believe the generality and the simplicity of our framework will be useful in studying various biomolecular interactions and provide novel insights into their dynamics.

## Author contributions

S.S. designed research, performed research, analyzed data, and wrote the paper. D.G. designed research, performed research, analyzed data, and wrote the paper.

## Supporting material

Document S1. Figures S1–S7

Document S2. Article plus supporting material
