# Supplementary material for: Lifetime of actin-dependent protein nanoclusters
Source: Biophys J. 2022 Dec 14;122(2):290–300. doi: 10.1016/j.bpj.2022.12.015 (PMC9892618; doi:10.1016/j.bpj.2022.12.015)
Supplement: Document S1. Figures S1–S7 [file mmc1.pdf]

Biophysical Journal, Volume *122*

Supplemental information

Lifetime of actin-dependent protein nanoclusters

Sumantra Sarkar and Debanjan Goswami

Supplementary Information: Lifetime of actin-dependent protein nanoclusters

Sumantra Sarkar[a,b,c] and Debanjan Goswami[d]

a The Center for Nonlinear Studies, Los Alamos National Laboratory, Los Alamos, New Mexico, USA, 87544
b Theoretical Biophysics (T-6) group, Los Alamos National Laboratory, Los Alamos, New Mexico, USA, 87544
c Present address: Department of Physics, Indian Institute of Science, Bangalore, India, 560012
d NCI RAS Initiative, The Cancer Research Technology Program, Frederick National Laboratory, Frederick, MD 21701

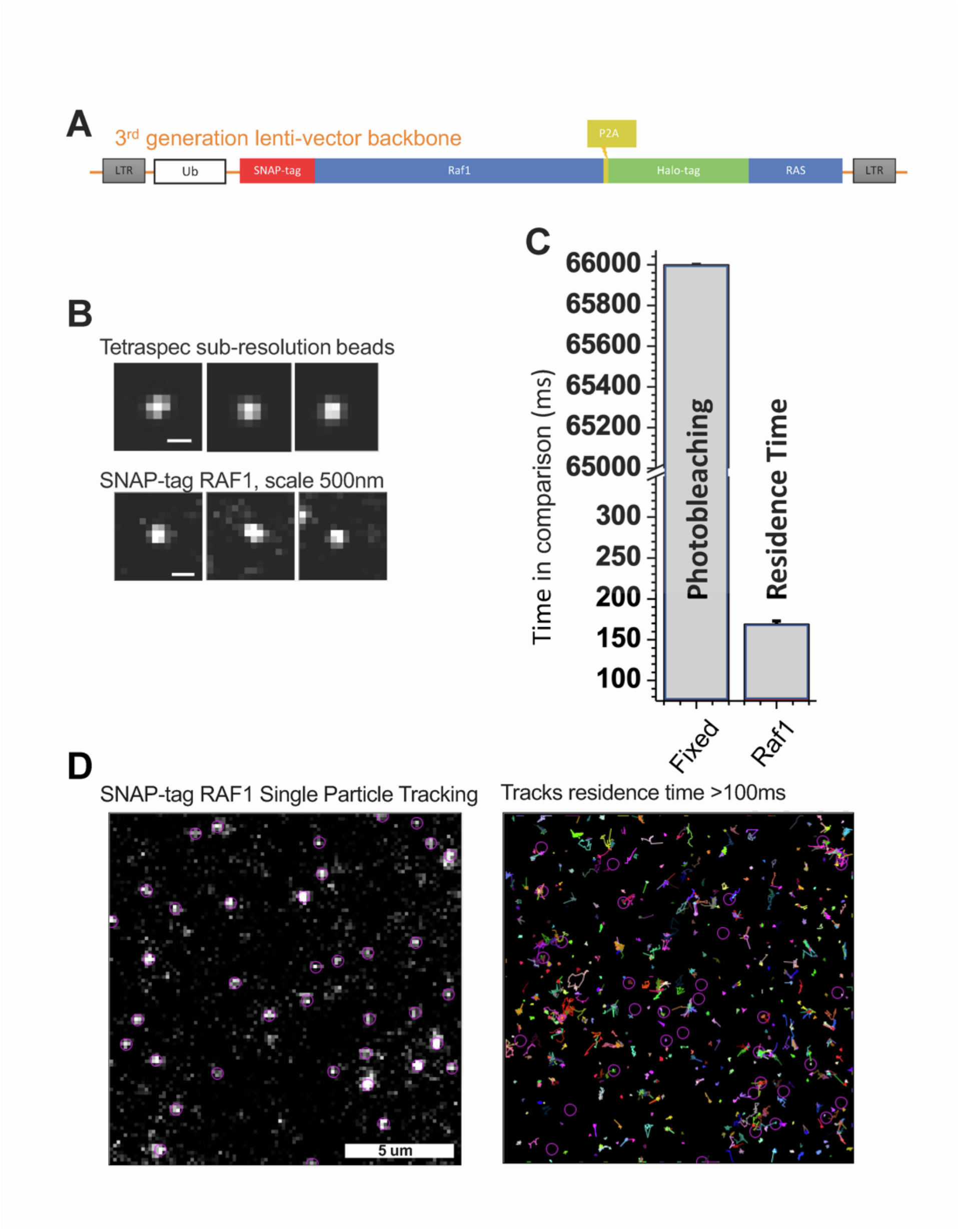

Figure S1. Experimental details: (A) Schematic of a 3rd generation lentiviral backbone where SNAP-tag Raf1 and Halo-tag Ras bicistronic (separated by a P2A sequence) mammalian expression construct was engineered. This vector was used for transduction in RAS-less, Raf-less MEF cells for expression of SNAP-tag Raf1 and Halo-tag Ras in 1:1 stoichiometric fashion after cleavage of P2A sequence by endogenous cellular enzyme. (B) Panel shows images of tetraspec sub-resolution beads under the TIRF objective and single molecules of 647-SiR dye-labeled SNAPtag Raf1 with comparable PSF. Validation of single molecule imaging. In our previous publication, we have verified single molecule imaging by step photobleaching (not shown here again). (C) SNAP-Cell ® 647-SiR dye-labeled fixed cells were exposed to prolonged acquisition time to determine the average photobleaching time. Same laser power was during all experiments. It shows roughly three orders of magnitude difference in residence time causing miniscule effect on residence time. (D) Example of a single frame from a movie is presented to show how single molecules of Raf1 appeared in microscopic images and were tracked by the trackmate software. In the right panel, tracks are presented from the single molecule tracking movie from the left panel. Scale bar is printed on each image panel.

Binding kinetics

Consider the binding of a protein P to the actin cytoskeleton A:

$$P + A \rightarrow PA$$
$$PA \rightarrow P + A$$

The first reaction occurs with a rate k_{on} and the second one with a rate k_{off}. Therefore, in chemical equilibrium, we have:

$$k_{on}[P][A] = k_{off}[PA]$$

Where $[X]$ denotes the concentration of the species X. The probability to find the molecule P in the bound state PA is, that is the binding probability is:

$$p_{bound} = \frac{[PA]}{[PA]+[P]} = \frac{k_{on}[A]}{k_{off}+k_{on}[A]} = \frac{k_{on}C}{k_{off}+k_{on}C}$$

If C remains unchanged then we can absorb it into the k_{on}, such that:

$$p_{bound} = \frac{k_{on}}{k_{off}+k_{on}} = \phi_d$$

Lifetime distribution from the model

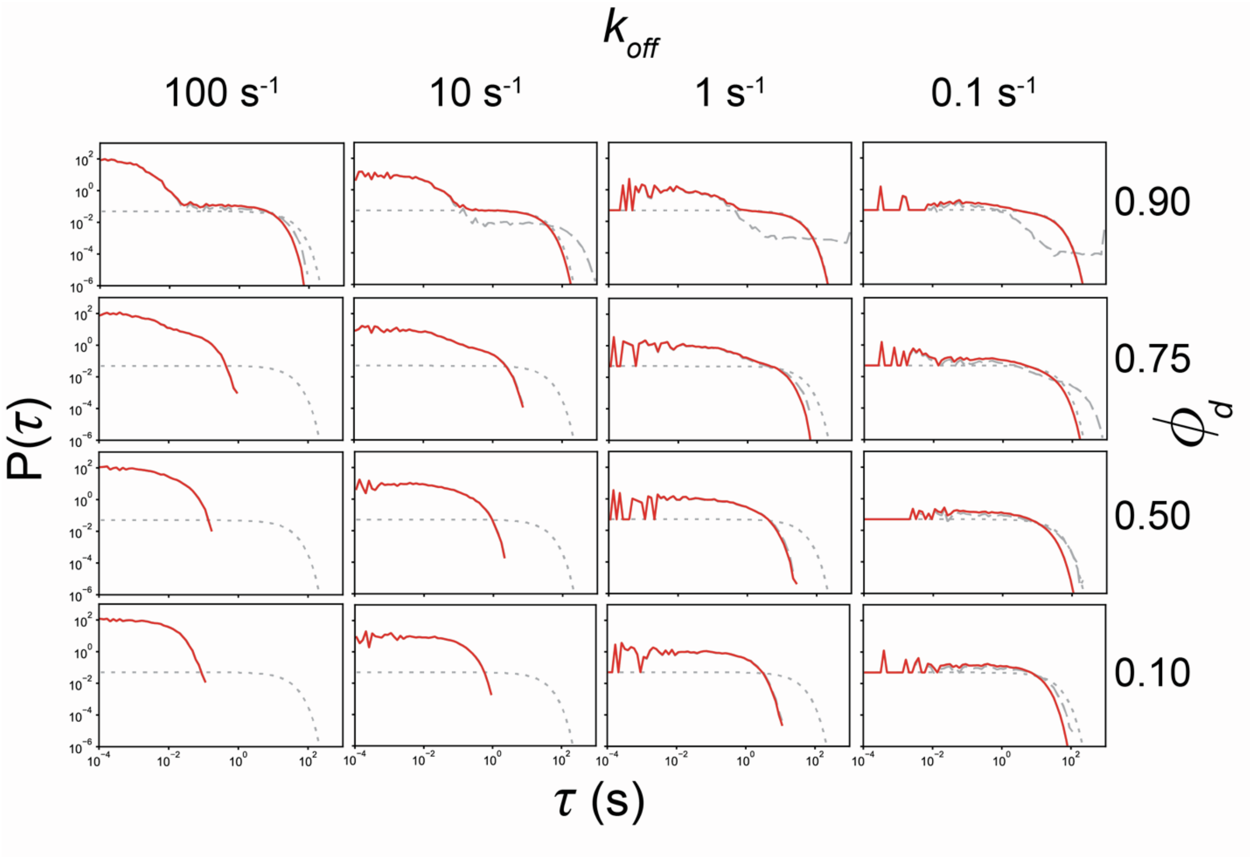

Figure S2. The lifetime distribution of protein adsorbed on actin aster. The aster lifetime τ_A has exponential distribution, $P_A(\tau_A)$with mean lifetime of $20\,s$ (grey dotted line). The desorption lifetime distribution $P_D(\tau_D)$ is shown using a gray dashed line and the cluster lifetime distribution, $P_C(\tau_C)$ is shown in red.

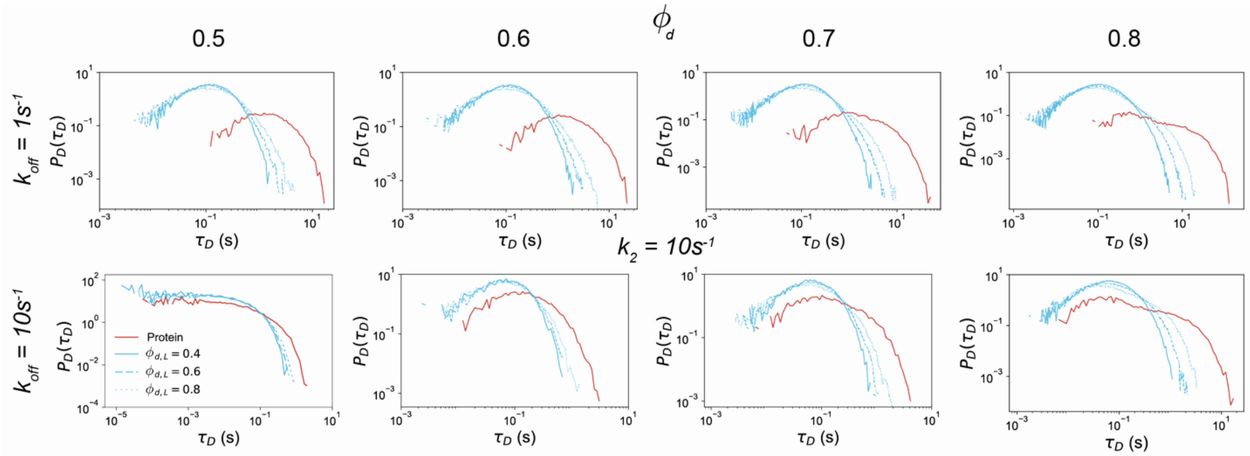

Figure S3. Desorption time distribution $P_D(\tau_D)$ of proteins (red) and ligands (sky-blue) when $N_{max} \geq 2$ and $M_{max} \geq 2$. The parameter values are shown in the figure.

Power law distribution of cluster lifetime

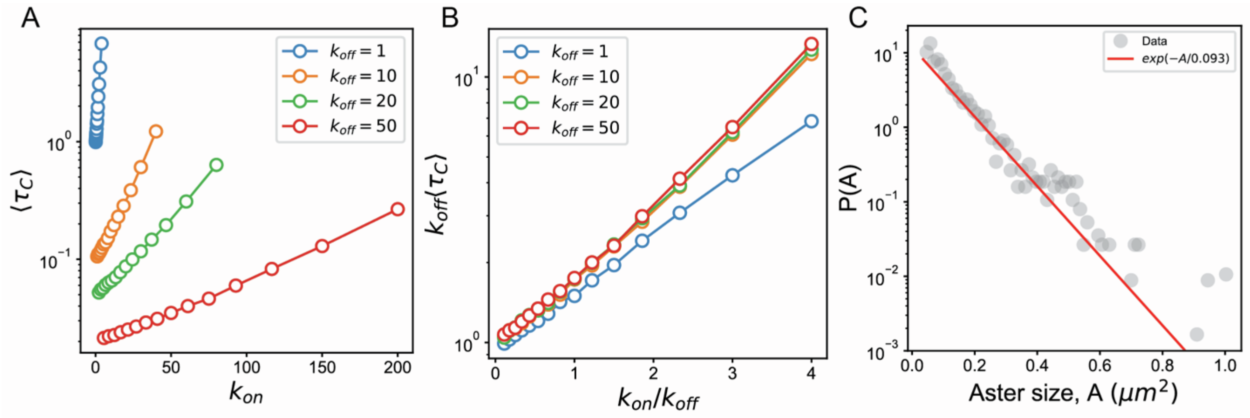

Figure S4.(A) Average cluster lifetime $\langle \tau_C \rangle$ vs k_{on} shows exponential dependence. (B) Scaling plot: $k_{off}\langle \tau_C \rangle$ vs k_{on}/k_{off} collapses curves at different k_{off} on top of each other for $k_{off} > 1$. Therefore $\langle \tau_C \rangle \sim k_{off}^{-1}\, e^{\frac{k_{on}}{k_{off}}}$, (C) The area of actin asters follow an exponential distribution, which implies that the concentration of actin also follows an exponential distribution. Data for (C) was previously published in [1]. The scale of the exponential distribution (0.093) is the reported median value of the aster sizes.

As figure S4A shows, the average cluster lifetime, $\langle \tau_C \rangle$ is an exponential function of k_{on}. In fact, as shown by the scaling of $\langle \tau_C \rangle$ in Fig. S4B

$$\langle \tau_C \rangle \sim k_{off}^{-1}\, e^{\frac{k_{on}}{k_{off}}}$$

Distribution of k_{on}

We have assumed that k_{on} is exponentially distributed. However, k_{on} is actually $\kappa_{on} C$, where C is the concentration of actin fiber at an aster and κ_{on} is a concentration independent constant. Therefore, for k_{on} to be exponentially distributed, C must be exponentially distributed. Experiments done by Xia et.al. shows that actin aster size indeed has an exponential distribution or at least a distribution with exponential tail (Fig. S4 C). Hence, we are justified in using an exponential distribution for k_{on}. In particular, we

have found that an exponential distribution with $\langle k_{on} \rangle = 30s^{-1}$ and $k_{off} = 20s^{-1}$ best fits the RAS residence time distribution (Fig 4A & S5).

Power law exponent

As predicted by Monthus and Bouchaud [2], if $\tau = e^{E/E_1}$ and $P(E) = \frac{1}{E_0} e^{-E/E_0}$, then $P(\tau) \propto \tau^{-1-\frac{E_1}{E_0}}$. Adapting this result to our case, we see that, $E = k_{on},\ E_0 = 30$ and $E_1 = 20$. Therefore, $P(\tau_C) \sim \tau_C^{-1.67}$, which shows remarkable agreement with the experimental results (Fig 4A).

Random Distribution of k_1

The distribution chosen for k_1 is an Weibull distribution with shape parameter 3 and scale parameter determined from $\langle k_1 \rangle$. The physical/biological justification of this choice is not as straightforward as for k_{on}. There is hardly any data available on how the free energy landscape of protein-protein interaction changes as lipid environment is varied. Therefore, it is difficult to justify this choice from biological literature. In contrast, in theories of spin-glasses it has been established that that the ground state free energy barriers follow Gumbel distribution, which leads to an Weibull distribution of reaction rates [3]. Such distribution of free energy barriers arises because of random interaction between neighboring spins. Translating this justification in the context of protein-protein interaction would imply that protein-protein interaction is disordered and has a rugged landscape. Indeed, in the context of RAS-RAF interaction, it is known that RAS and the Ras-binding-domain (RBD) of RAF interact differently depending on their orientation, which, in turn, can be modified by the lipid environment [4,5].

One of our key conclusions that WT and G12D Ras have identical binding affinity with Raf does not depend on the choice of k_{on} and k_1. Our theory predicts that the residence time distributions collapses on top of each other only when the binding affinity are identical. This prediction remains valid even when k_{on} and k_1 are not randomly distributed. To fit the experimental data with our model's prediction, we had to assume random distributions of k_{on} and k_1. However, the prediction remains valid even before fitting.

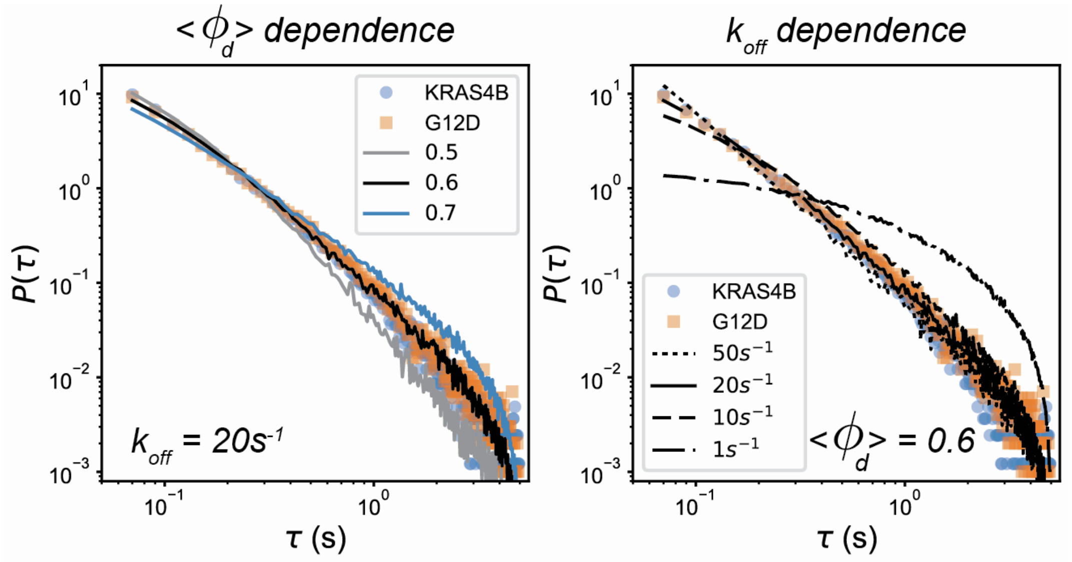

Fig S5. Fitting the model predictions of PNC lifetime distribution for various values of $\langle K_d \rangle$ for $k_{off} = 20\ s^{-1}$ (left) and k_{off} for $\langle K_D \rangle$ (right), which shows that the theoretical curves best fit the experimental data when $\langle K_D \rangle = 0.6$ and $k_{off} = 20s^{-1}$.

Rebinding of a single ligand: a chemical kinetic model

A ligand molecule may rebind to a protein target after dissociating from the target. The rebinding of the ligand enhances its lifetime near the protein. This effect is enhanced near protein nanoclusters, where the ligand can find a large number of targets to rebind before diffusing away into the bulk. Therefore, the residence time distribution of a ligand can change drastically near a protein nanocluster.

To understand the effect of rebinding on the residence time distribution of a single ligand molecule, we consider the following reactions:

1. Free ligand entering a region (reaction domain) where it can interact with the proteins in the nanocluster.
$$\phi \xrightarrow{k_{cm}} F \cdots (S1)$$
2. Free ligand diffusing into the bulk.
$$F \xrightarrow{k_{mc}} \phi \cdots (S2)$$
3. Free ligand interacting with the protein target to form ligand-protein complex.
$$F + P \overset{k_1}{\rightarrow} PR \cdots (S3)$$
4. Ligand-protein complex dissociating to produce a free ligand.
$$PR \overset{k_2}{\rightarrow} F + P \cdots (S4)$$
5. Ligand-protein complex dissociating from the membrane and diffusing to the bulk.
$$PR \xrightarrow{k_{off}} \phi \cdots (S5)$$

In addition, the protein nanocluster dynamics is governed by the reactions described in Fig 1A. To ensure that there is only one ligand in the reaction domain, reactions 1 and 4 can occur only when $F = 0$ and reactions 2 and 3 can only occur when $F = 1$. As a result, the ligand can bind to / unbind from the protein multiple times before escaping to the bulk.

We estimate k_{cm} by finding the diffusion limited rates of binding to an absorbing spherical domain of radius a, which is the radius of the reaction domain. Because the reaction domain is hemispherical, k_{cm} will be proportional to the value for a spherical domain. The absorption rate of a spherical domain depends on the bulk concentrations, c_∞, of the ligands and their diffusivities, D. For purely diffusive transport, k_{cm} will be proportional to $4\pi D a c_\infty$, where a is the radius of the hemispherical reaction domain [6]. For $c_\infty = 200/\mu m^3$, $D = 10\ \mu m^2/s$, and $a \approx 5 - 50\ nm = 0.005 - 0.05\ \mu m$ which are the typical numbers for signaling protein clusters and their protein partners, $k \approx 100 - 1000/s$.

Similarly, the escape rate k_{mc} can be approximated from the first escape time, τ, from a spherical domain of radius a, for which the escape time distribution is given by [7,8]:

$$q(\tau) = -2\sum_{n=1}^{\infty}(-1)^n \exp\left(-\frac{n^2\pi^2}{a^2}Dt\right)\frac{n^2\pi^2 D}{a^2} \cdots (S6)$$

From this distribution, it is easy to compute the mean escape time, which for the same values of D and a is around $10^{-5} - 10^{-4}s$, which implies that $k_{mc} \approx 10^4 - 10^5/s$. The residence time distribution of the ligand for these values of k_{mc} and k_{cm} are shown in the following figure. Clearly, the residence time does not show any nonmonotonic behavior.

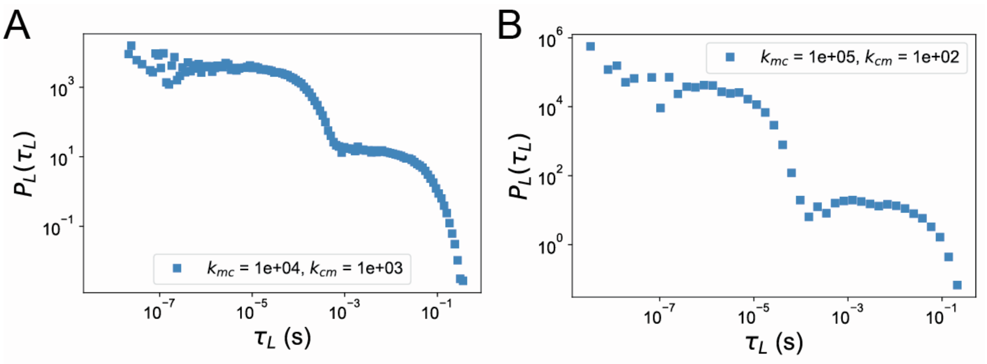

Fig S6. Residence time distribution of single ligand for biologically relevant parameter values does not show nonmonotonic distributions. $\langle K_d \rangle = 0.6$, $k_{off} = 20s^{-1}$, $\langle K_{d,L} \rangle = 0.6$, $k_2 = 10s^{-1}$. k_{on} is distributed as an exponential and k_1 is distributed as a Weibull distribution. In both cases the mean values are determined by the mean duty ratios and the dissociation rates.

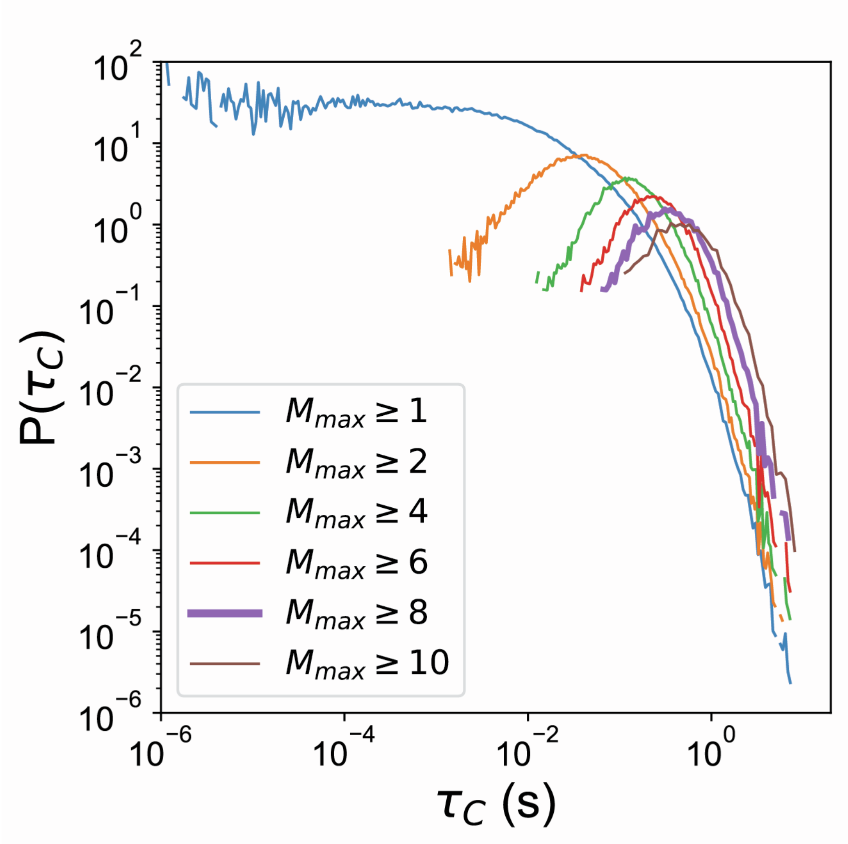

Figure S7. The cluster lifetime distribution $P(\tau_C)$ of the ligand (RAF) when k_1 is distributed as a Weibull distribution with shape parameter 3 and mean determined by $\langle\phi_{d,L}\rangle$ = 0.6 and $k_2 = 10s^{-1}$. We plot the distributions of τ_C for $M_{max} \geq M_{th}$. The values of M_{th} are shown in the legend. For this plot $k_{off} = 20s^{-1}$, $\langle\phi_d\rangle = 0.6$, and that k_{on} is distributed as a Weibull distribution with shape parameter 1 (exponential distribution).

[1] S. Xia, Y. B. Lim, Z. Zhang, Y. Wang, S. Zhang, C. T. Lim, E. K. F. Yim, and P. Kanchanawong, *Nanoscale Architecture of the Cortical Actin Cytoskeleton in Embryonic Stem Cells*, Cell Reports **28**, 1251 (2019).

[2] C. Monthus and J.-P. Bouchaud, *Models of Traps and Glass Phenomenology*, J. Phys. A: Math. Gen. **29**, 3847 (1996).

[3] M. Körner, H. G. Katzgraber, and A. K. Hartmann, *Probing Tails of Energy Distributions Using Importance-Sampling in the Disorder with a Guiding Function*, J. Stat. Mech. **2006**, P04005 (2006).

[4] V. A. Ngo, S. Sarkar, C. Neale, and A. E. Garcia, *How Anionic Lipids Affect Spatiotemporal Properties of KRAS4B on Model Membranes*, J. Phys. Chem. B (2020).

[5] T. Travers, C. A. Lopez, Q. N. Van, C. Neale, M. Tonelli, A. G. Stephen, and S. Gnanakaran, *Molecular Recognition of RAS/RAF Complex at the Membrane: Role of RAF Cysteine-Rich Domain*, Scientific Reports **8**, 8461 (2018).

[6] H. C. Berg and E. M. Purcell, *Physics of Chemoreception*, Biophysical Journal **20**, 193 (1977).

[7] L. Sbailò and F. Noé, *An Efficient Multi-Scale Green's Function Reaction Dynamics Scheme*, J Chem Phys **147**, 184106 (2017).

[8] T. R. Sokolowski, J. Paijmans, L. Bossen, T. Miedema, M. Wehrens, N. B. Becker, K. Kaizu, K. Takahashi, M. Dogterom, and P. R. ten Wolde, *EGFRD in All Dimensions*, J. Chem. Phys. **150**, 054108 (2019).
